# Extracts of six Rubiaceae species combined with rifampicin have good in vitro synergistic antimycobacterial activity and good anti-inflammatory and antioxidant activities

**DOI:** 10.1186/s12906-016-1355-y

**Published:** 2016-10-03

**Authors:** Abimbola O. Aro, Jean P. Dzoyem, Jacobus N. Eloff, Lyndy J. McGaw

**Affiliations:** 1 Phytomedicine Programme, Department of Paraclinical Sciences, Faculty of Veterinary Science, University of Pretoria, Private Bag X04, Onderstepoort, 0110 South Africa; 2 Present Address: Department of Biochemistry, Faculty of Science, University of Dschang, P.O. Box 67, Dschang, Cameroon

**Keywords:** Synergy, Antimycobacterial, Antioxidant, Nitric oxide, Rubiaceae, Tuberculosis

## Abstract

**Background:**

The Rubiaceae family has played a significant role in drug discovery by providing molecules with potential use as templates for the development of therapeutic drugs. This study was designed to study the in vitro synergistic effect of six Rubiaceae species in combination with a known anti-TB drug. The antioxidant and anti-inflammatory activity of these species were also evaluated to investigate additional benefits in antimycobacterial treatment.

**Methods:**

The checkerboard method was used to determine the antimycobacterial synergistic activity of plant extracts combined with rifampicin. The antioxidant activity of extracts was determined by the 2,2-diphenyl-1-picrylhydrazyl (DPPH) method. Anti-inflammatory activity via inhibition of nitric oxide (NO) production was performed in LPS-activated RAW 264.7 macrophages using the Griess assay.

**Results:**

Combination of rifampicin with the crude extracts resulted in a 4 to 256-fold increase of activity of extracts. The crude extract of *Cremaspora triflora* produced the best synergistic effect with rifampicin, with a fractional inhibitory concentration (FIC) index of 0.08 against *Mycobacterium aurum*. Extracts of *Psychotria zombamontana* had the best antioxidant activity with an IC_50_ value of 1.77 μg/mL, lower than the IC_50_ of trolox and ascorbic acid (5.67 μg/mL and 4.66 μg/mL respectively). All the extracts tested inhibited nitric oxide (NO) production in a concentration dependent manner with the percentage of inhibition varying from 6.73 to 86.27 %.

**Conclusion:**

Some of the Rubiaceae species investigated have substantial in vitro synergistic effects with rifampicin and also good free radical scavenging ability and anti-inflammatory activity. These preliminary results warrant further study on these plants to determine if compounds isolated from these species could lead to the development of bioactive compounds that can potentiate the activity of rifampicin even against resistant mycobacteria.

## Background

The emergence of drug resistant tuberculosis (TB) strains (including multi drug resistant, MDR and extremely drug resistant, XDR) has reduced the efficacy of first-line anti-TB drugs. Therefore, there is an urgent need to design drugs that can act in synergy with previously potent anti-TB drugs. The implementation of combination therapy is of great importance for the treatment of infectious diseases, as monotherapy is often associated with an increase in the development of resistance. In order to improve the cure rate of infectious disease and curb the upsurge of resistance, combination therapy is encouraged, as has been shown with the multidrug approach used to treat tuberculosis (Isoniazid, Rifampicin, Pyrazinamide and Ethambutol). Without such combination therapy, the death rate of infected patients could have reached global epidemic proportions [1].

Most in vitro studies determine the therapeutic effect of new drugs as single agents, but in clinical practice to prevent the development of drug resistance, a combination of various drugs is imperative [2]. Combination studies with natural products from plants and synthetic drugs are limited to relatively few reports. Therefore, evaluating the in vitro effect of bioactive extracts or compounds with the classic anti-TB drugs is necessary and may lead to more effective treatment of TB [3, 4].

One of the manifestations of inflammation is oxidative stress, and the pathways that generate the mediators of inflammation are all induced by oxidative stress [5]. Most of the potentially harmful effects of oxygen are due to the formation of reactive oxygen species (ROS). The role played by the reactive oxygen intermediates during TB infection is not fully understood, but it is known that hydrogen peroxide produced by macrophages which have been activated by cytokines has mycobactericidal activity [6]. Excess reactive oxygen species (ROS) generated could however, lead to inflammation, and antioxidant substances that can scavenge and eliminate ROS may be useful in preventing or minimizing oxidation-related diseases such as TB [7]. Costantino et al. [8] reported several studies showing the correlation between antioxidant properties of plants with oxidative stress defence.

Nitric oxide (NO) plays a significant role during the host defence response against different pathological organisms such as bacteria, viruses, parasites and fungi [9]. NO is important in regulating diverse pathophysiological process in the body under normal physiological conditions [10]. It is a free radical involved in many biological processes, with the ability to enhance the bactericidal activity of activated macrophages [11]. In case of treatment failure or of infection with resistant strains, mycobacteria persist in the macrophages of the host for a long period. This can lead to an overexpression of inflammatory mediators including nitric oxide and further generate oxidative stress due to an imbalance between pro-oxidant and antioxidant systems. Furtermore, excessive production of NO may lead to potential tissue damage associated with acute and chronic inflammation [12]. Many researchers have demonstrated the potential of medicinal plant extracts to scavenge free radicals and modulate inflammatory reactions [13, 14]. The mammalian immune system combats pathogens through activated macrophages, thereby providing microbicidal activity before the initiation of various pro-inflammatory mediators such as NO [10]., Lipopolysaccharide (LPS) a Gram-negative bacterial cell wall component is a good activator of macrophages, and is also involved in the production of pro-inflammatory cytokines [15]. Therefore, much attention is being given to the invention of drugs which can act as potent inhibitors of NO production (such as that detected in LPS stimulated RAW 264.7 cells) as a possible treatment for chronic inflammatory disease [16]. Plant products have been used in the development of new drugs, and they continue to play an important role in the discovery and development of new drugs [17]. Therefore, plant products that can inhibit the production of NO may be a potential therapy for those diseases in which inflammation plays a role [18].

Various species from the Rubiaceae family have proven to be a promising source of new bioactive substances which may lead to the development of new products as active molecules or drug prototypes due to their structural diversity and pharmacological activities [19]. One peculiar characteristic of this family is that they contain a wide range of secondary metabolites such as alkaloids, flavonoids, terpenes, anthraquinones and coumarins having good pharmacological properties [20]. These classes of secondary metabolites have been associated with antimicrobial, anti-malarial, hepatoprotective, antioxidant, and many other interesting biological activities [21].

In a previous study, six species from the Rubiaceae family, namely *Cephalanthus natalensis*, *Cremaspora triflora, Oxyanthus speciosus*, *Pavetta lanceolata*, *Psychotria capensis* and *Psychotria zombamontana* were shown to have good antimycobacterial activity [22]. These species were selected for further study to determine their synergistic activity with a known antimycobacterial drug., The antioxidant and nitric oxide inhibitory properties were also determined in an effort to gain more insight into their potential mechanism of action.

## Methods

### Plant collection

The leaves of plants were collected in the Pretoria National Botanical Garden in terms of a signed material transfer agreement. The plant materials were identified by the curator and labelled. Voucher specimens were kept in the HGWJ Schweickerdt Herbarium of the University of Pretoria (PRU) (Table 1). The leaves were air-dried at room temperature, ground to a fine powder in a Macsalab mill (Model 200 LAB, Eriez, Bramley, Johannesburg, South Africa) and stored in closed glass containers in the dark until needed. Acetone was selected as the extracting solvent for the dried leaf powder owing to its ability to extract a wide range of compounds and its low toxicity in the antimicrobial bioassay [23]. Ground leaf material was extracted by acetone in a ratio of 10:1 (v:m), evaporated to dryness and made up to a concentration of 10 mg/mL.

**Table 1:** Synergistic activity of extracts from six Rubiaceae species and rifampicin against *Mycobacterium* species

Extract/rifampicin	Mycobacterial strains	MIC (μg/ml)		FIC^a^		Interpretation
		Individual^b^	Combination^c^	Individual	Index	
*Cephalanthus natalensis*/rifampicin	*M. smegmatis*	17/16	0.531/8	0.03/0.5	0.53	Synergy
			0.133/8	0.008/0.5	0.51	Synergy
			0.066/8	0.004/0.5	0.5	Synergy
	*M. aurum*	100/78	50/1.21	0.5/0.02	0.52	Synergy
			50/9.75	0.5/0.13	0.63	Additive
			1.56/19.5	0.02/0.25	0.27	Synergy
	*M. tuberculosis*	40/9	40/1.125	1/0.125	1.125	Non-interactive
			5/2.25	0.125/0.25	0.38	Synergy
			2.5/2.25	0.063/0.25	0.31	Synergy
*Cremaspora triflora*/rifampicin	*M. smegmatis*	23/16	11.5/8	0.5/0.5	1	Additive
			5.7/8	0.25/0.5	0.75	Additive
			2.88/8	0.125/0.5	0.63	Additive
	*M. aurum*	100/78	6.25/1.21	0.063/0.016	0.08	Synergy
			50/2.43	0.5/0.031	0.53	Synergy
			25/4.87	0.25/0.063	0.31	Synergy
	*M. tuberculosis*	160/9	40/0.14	0.25/0.016	0.27	Synergy
			20/0.56	0.125/0.062	0.19	Synergy
			10/1.125	0.062/0.125	0.19	Synergy
*Oxyanthus speciosus*/rifampicin	*M. smegmatis*	80/16	20/8	0.25/0.5	0.75	Additive
			10/8	0.125/0.5	0.63	Additive
			5/8	0.06/0.5	0.56	Additive
	*M. aurum*	60/78	60/78	1/1	2	Non-interactive
			30/78	0.5/1	1.5	Non-interactive
			15/78	0.25/1	1.25	Non-interactive
	*M. tuberculosis*	170/9	85/2.25	0.5/0.25	0.75	Additive
			21.25/2.25	0.125/0.25	0..38	Synergy
			5.31/2.25	0.031/0.25	0.28	Synergy
*Pavetta lanceolata*/rifampicin	*M. smegmatis*	12/16	6/8	0.5/0.5	1	Additive
			3/8	0.25/0.5	0.75	Additive
			1.5/8	0.125/0.5	0.63	Additive
	*M. aurum*	100/78	6.25/39	0.063/0.5	0.56	Additive
			0.781/39	0.008/0.5	0.51	Synergy
			0.391/39	0.004/0.5	0.5	Synergy
	*M. tuberculosis*	120/9	60/2.25	0.5/0.25	0.75	Additive
			30/4.5	0.25/0.5	0.75	Additive
			7.5/4.5	0.063/.05	0.56	Additive
*Psychotria capensis*/rifampicin	*M. smegmatis*	60/16	30/8	0.5/0.5	1	Additive
			15/8	0.25/0.5	0.75	Additive
			7.5/8	0.125/0.5	0.63	Additive
	*M. aurum*	120/78	30/19.5	0.25/0.25	0.5	Synergy
			15/39	0.13/0.5	0.63	Additive
			3.75/39	0.03/0.5	0.53	Synergy
	*M. tuberculosis*	630/9	315/4.5	0.5/0.5	1	Additive
			157.5/4.5	0.25/0.5	0.75	Additive
			78.75/4.5	0.125/0.5	0.63	Additive
*Psychotria zombamontana*/rifampicin	*M. smegmatis*	40/16	10/8	0.25/0.5	0.75	Additive
			5/8	0.125/0.5	0.63	Additive
			2.5/8	0.06/0.5	0.56	Additive
	*M. aurum*	80/78	20/19.5	0.25/0.25	0.5	Synergy
			10/19.5	0.125/0.25	0.38	Synergy
			5/19.5	0.063/0.25	0.31	Synergy
	*M. tuberculosis*	160/9	80/2.25	0.5/0.25	0.75	Additive
			40/2.25	0.25/0.25	0.5	Synergy
			20/2.25	0.125/0.25	0.38	Synergy

^*a*^ *FIC* fractional inhibitory concentration for individual sample FIC index + = FIC (a) + FIC (b). ^b^ *MIC* minimum inhibitory concentration for individual sample; ^c^MIC of samples in combination

### Mycobacterial culture

Antimycobacterial activity was tested against two non-tuberculous mycobacteria (NTM): *Mycobacterium smegmatis* (ATCC 1441) and *Mycobacterium aurum* (NCTC 10437), as well as against a *M. tuberculosis* field strain (TB 8104) which was isolated from a waterbuck and genetically characterized using variable number of tandem repeat typing based on a 13-locus VNTR panel [24].

### In vitro synergistic antimycobacterial activity

The effect of combinations of the acetone extracts of *Cremaspora triflora, Cephalanthus natalensis*, *Oxyanthus speciosus, Pavetta lanceolata*, *Psychotria capensis* and *Psychotria zombamontana* with rifampicin (RIF, Sigma) against three mycobacteria were determined using the checkerboard method [25]. Rifampicin was dissolved in DMSO (0.25 %). The Fractional Inhibitory Concentration (FIC) of each drug was calculated by dividing the concentration of the extract present in that well in combination, where complete inhibition of growth of the microorganism was observed, by the MIC of that extract alone to inhibit the microorganism. All assays were performed in triplicate. The ΣFIC was then calculated for each test sample independently as specified in the following equations:

FIC (a) = MIC (a) in combination with (b)/ MIC (a) alone

FIC (b) = MIC (b) in combination with (a)/ MIC (b) alone

The sum of the FIC or FIC index is thus calculated as: ΣFIC = FIC (a) + FIC (b)

The interpretations of the ΣFICs were as follows: ΣFIC ≤ 0.5 = synergistic; >0.5–1.0 = additive; >1.0–≤4.00 = non-interactive and >4.0 = antagonistic [1].

### DPPH assay

The DPPH radical-scavenging activity was determined as previously described [7]. Ascorbic acid and trolox were used as positive controls, methanol as negative control and extract without DPPH as blank. Results were expressed as percentage reduction of the initial DPPH absorption in relation to the control. The concentration of extract leading to 50 % reduction of DPPH (IC_50_) was also determined.

### Inhibition of nitric oxide (NO) production

The NO production inhibitory activity of plant extracts was evaluated in the LPS-activated mouse macrophage cell line RAW 264.7 as previously described [7]. Validity of the assays was shown by using untreated cells as negative control, LPS-stimulated cells as positive control and additionally a cell group as reduction control group with LPS-stimulated cells, co-incubated together with quercetin used as an inhibitor of NO.

### Statistical analysis

All experiments were conducted in triplicate and values expressed as mean ± standard deviation. Statistical analysis was performed using one way ANOVA and results were compared using the Fisher’s least significant difference (LSD) at a 5 % significance level.

## Results

The results of the combination of the six crude extracts with rifampicin against *M. smegmatis*, *M. aurum* and *M. tuberculosis* (8104) showed varying degrees of synergistic to non-interactive antimycobacterial effects. Upon addition of sub-minimal inhibitory concentrations of the acetone crude extracts to rifampicin, reduction of the MICs of the anti-TB drug ranged from 2-fold to 4-fold (ΣFIC = 0.5–1), 2-fold to 64-fold (ΣFIC = 0.08–2) and 2-fold to 64-fold (ΣFIC =0.19–1) for *M. smegmatis*, *M. aurum* and *M. tuberculosis* respectively (Table 1). The MIC values of the crude extracts against the three tested mycobacteria were reduced to varying degrees. *Cremaspora triflora* and *Pavetta lanceolata* extract MIC values were decreased 16-fold to 256-fold in combination with rifampicin (ΣFIC = 0.08–0.5) against *M. aurum*. The crude extract of *Pavetta lanceolata* also showed some synergistic to additive effect.

Regarding antioxidant activity, plant extracts with IC_50_ < 50 μg/mL are considered to have significant antioxidant activity while samples with IC_50_ > 50 μg/mL are classified as having moderate antioxidant activity [26]. Compounds with IC_50_ < 10 μg/mL are considered to have significant antioxidant capacity while 10 < IC_50_ < 20 μg/mL are considered moderate and IC_50_ > 20 μg/mL are said to possess low antioxidant activity [27]. The lower the IC_50_ value of an extract, the higher its antioxidant activity. The crude extract of *Psychotria zombamontana* had the best antioxidant activity among the six tested extracts with an IC_50_ value of 1.77 μg/mL compared to trolox and ascorbic acid (with IC_50_ values of 5.67 μg/mL and 4.66 μg/mL respectively). Based on the above cut-off criteria, *Psychotria capensis* also had good antioxidant activity with an IC_50_ value of 29.73 μg/mL while other extracts had moderate to weak antioxidant capacity (IC_50_ values ranging from 68.64–259.7 μg/mL).

As shown in Table 2, all the extracts had a dose dependent inhibition of NO production at concentrations of 6.25, 12.5, 25 and 50 mg/mL. The crude acetone extract of *Psychotria capensis* had the best inhibitory activity on NO production (86.27 %), but this is most likely due to cytotoxic effects of the extract on the RAW 264.7 macrophage cell line. Percentage cell viability at 50 and 25 μg/mL respectively was as low as 4.44 % and 28.31 %. The acetone extract of *Psychotria capensis* has been reported for its cytotoxic effect on C3A human liver and Vero kidney cells [22]. *Pavetta lanceolata* also had good anti-inflammatory activity by inhibiting the production of NO by 70 % with no concomitant cytotoxic effect on RAW 264.7 macrophages.

**Table 2:** Antioxidant and NO production inhibitory activity and cell viability of extracts from six Rubiaceae species

Samples	Concentration (μg/mL)	NO (μM)	% NO inhibition	Cell viability	DPPH (IC_50_ in μg/mL)	Voucher no
*Cephalanthus natalensis*	50	1.26 ± 0.14	53.81	104.67	68.64 ± 0.20^a^	PRU 120872
	25	1.32 ± 0.09	51.39	100.84		
	12.5	1.80 ± 0.08	33.95	90.02		
	6.25	2.34 ± 0.06	14.10	76.19		
*Cremaspora triflora*	50	1.97 ± 0.16	27.66	96.70	90.02 ± 0.10^b^	114710
	25	2.43 ± 0.13	10.70	105.20		
	12.5	2.48 ± 0.08	8.77	104.25		
	6.25	2.90 ± 0.06	6.73	118.31		
*Oxyanthus speciosus*	50	1.63 ± 0.06	40.25	109.84	259.7 ± 0.23^c^	PRU 120873
	25	1.57 ± 0.07	42.19	97.04		
	12.5	1.77 ± 0.12	34.92	91.61		
	6.25	1.94 ± 0.15	28.63	88.82		
*Pavetta lanceolata*	50	0.80 ± 0.16	70.77	101.73	138.94 ± 0.18^d^	PRU 120874
	25	1.26 ± 0.10	53.81	112.10		
	12.5	1.84 ± 0.13	32.50	116.49		
	6.25	2.52 ± 0.10	7.31	123.03		
*Psychotria capensis*	50	0.37 ± 0.05	86.27	4.44	29.73 ± 0.22^e^	PRU 120875
	25	1.08 ± 0.77	60.11	28.31		
	12.5	2.97 ± 0.22	9.16	75.40		
	6.25	3.22 ± 0.23	18.36	115.71		
*Psychotria zombamontana*	50	2.19 ± 0.10	19.42	114.53	1.77 ± 0.13^f^	PRU 120867
	25	2.02 ± 0.20	25.72	93.32		
	12.5	2.05 ± 0.14	24.75	91.92		
	6.25	2.40 ± 0.10	11.67	76.91		
Quercetin	25	0.35 ± 0.10	95.54	49.33	nd	
	12.5	0.30 ± 0.05	96.14	60.69		
	6.25	0.69 ± 0.08	91.08	73.76		
	3.12	2.50 ± 0.48	73.76	73.10		
Trolox	nd	nd	nd	nd	5.67 ± 0.24^f^	nd
Ascorbic acid	nd	nd	nd	nd	4.66 ± 0.25^h^	nd

Values with different letters are significantly different at *p* < 0.05

## Discussion

Spontaneous genetic mutations in *Mycobacterium tuberculosis* may lead to the emergence of drug resistant strains following inconsistent or inappropriate single drug therapy using selected anti-TB drugs [28]. Rifampicin is one of the most effective first-line TB drugs which is active against drug-susceptible TB, but the emergence of resistant strains is threatening the continued efficacy of this drug. Synergistic effects can occur if the constituents of an extract affect different targets or interact with one another in order to enhance the bioavailability of one or several substances of an extract [29]. The acetone extract of *Cremaspora triflora* in combination with rifampicin had the best fractional inhibitory concentration (ΣFIC = 0.08) showing a synergistic effect and leading to a 64-fold reduction in the MIC of rifampicin i.e. a 64-fold increase in the antimycobacterial activity. *Oxyanthus speciosus* in combination with rifampicin had a non-interactive effect (ΣFIC = 2) against *M. aurum*. Based on this assay, only a two-fold reduction in the MIC of rifampicin was observed against *M. smegmatis*. Encouragingly, no antagonist effect was observed using the different combinations against all the tested strains. Synergy in terms of natural product combinations may be affected by many factors such as: pharmacokinetics, physicochemical properties, complex multi-target effects or therapeutic approaches [29].

It has been reported that the antioxidant activity of plant materials is well correlated with the content of their phenolic compounds [30]. Different studies have also shown a positive correlation between phenolic compounds and terpene concentration with antimicrobial activity [31, 32].

Of the six extracts tested, *Psychotria zombamonatana* and *Psychotria capensis* had good antioxidant activity. Others had only moderate to weak free radical scavenging capacity, with *Oxyanthus speciosus* having the lowest antioxidant activity. Extracts with good inhibitory activity on NO production and low cytotoxicity are more useful for potential therapeutic application. Anti-inflammatory activity of plant extracts above 70 % at a concentration of 250 μg/ml is considered significant [33]. The six crude extracts screened in this study all led to dose-dependent NO inhibition. However, when compared with the activity of quercetin, the extracts did not meaningfully inhibit the production of NO, except for the acetone extract of *Psychotria capensis.* This extract had toxicity to the LPS-stimulated RAW 264.7 macrophage cell line, which limits its usefulness. The synergistic effect of many compounds in natural products enhances their antioxidant properties. The acetone extract of *Psychotria capensis* showed a synergistic antimycobacterial effect in combination with rifampicin and also had good antioxidant activity while inhibiting the production of NO.

Antioxidants act by scavenging free radicals such as reactive oxygen species, hydroxyl radicals and nitric oxide while anti-inflammatory mediators act by modulating the activities of pro-inflammatory enzymes and cytokines. In inflammation, nitric oxide (NO) acts as a pro-inflammatory mediator and is synthesized by inducible nitric oxide synthase (iNOS) in response to pro-inflammatory agents such as lipopolysaccharide (LPS) [34]. The inhibitory activity of NO production by medicinal plants may originate from the inhibition of iNOS enzyme activity and/or expression of nitric oxide synthase [35]. Many natural compounds from medicinal plants are known inhibitors of iNOS expression in LPS-activated macrophages [36]. No report could be found on the inhibitory activity of the Rubiaceae plant extracts tested in this study on NO synthesis in LPS-activated RAW 264.7 cells. The selected plant species have been reported for their antimycobacterial activities against mycobacteria but possess weak antioxidant and anti-inflammatory activity. Because of the role rifampicin plays in the treatment of TB, researchers are interested in compounds that can display synergistic activity with this efficacious anti-TB drug in order to increase both therapeutic efficacy and reduce toxicity commonly observed by this drug [37, 38].

Despite the weak antioxidant and anti-inflammatory activities, these plant extracts were able to decrease the MIC of rifampicin, thereby enhancing the mycobactericidal activity of this drug. The synergistic interaction observed in this study may be important, as rifampicin plays a pertinent role in reducing the duration of treatment for TB, thereby enhancing patients’ compliance to prevent the development of drug resistant cases. Furthermore, reducing the dosage of rifampicin by using different drug combinations could reduce adverse effects observed during administration of this antimycobacterial drug.

## Conclusion

This study investigated the in vitro synergistic activity of six Rubiaceae species in combination with the first-line antitubercular drug rifampicin. All the screened crude extracts produced synergistic to additive effects at different concentrations when combined with rifampicin but no antagonist effect was observed. A promising observation was the lack of cytotoxicity observed with some of the extracts. Whole animal studies are required to confirm the low toxicity of the extracts. The findings from this study are preliminary but are sufficient to warrant further study on these Rubiaceae species, as compounds isolated from these species could lead to the development of novel antimycobacterial compounds.

## Abbreviations

DPPH: 2,2-diphenyl-1-picrylhydrazyl
FIC: Fractional inhibitory concentration
iNOS: Inducible nitric oxide synthase
LPS: Lipopolysaccharide
MDR: Multi drug resistant
MIC: Minimum inhibitory concentration
NO: Nitric oxide
RIF: Rifampicin
ROS: Reactive oxygen species
TB: Tuberculosis
XDR: Extremely drug resistant
